# Sequence labeling via reinforcement learning with aggregate labels

**DOI:** 10.3389/frai.2024.1463164

**Published:** 2024-11-15

**Authors:** Marcel Geromel, Philipp Cimiano

**Affiliations:** Center for Cognitive Interaction Technology, Bielefeld University, Bielefeld, Germany

**Keywords:** reinforcement learning, reward functions, annotations, sequence labeling, information extraction

## Abstract

Sequence labeling is pervasive in natural language processing, encompassing tasks such as Named Entity Recognition, Question Answering, and Information Extraction. Traditionally, these tasks are addressed via supervised machine learning approaches. However, despite their success, these approaches are constrained by two key limitations: a common mismatch between the training and evaluation objective, and the resource-intensive acquisition of ground-truth token-level annotations. In this work, we introduce a novel reinforcement learning approach to sequence labeling that leverages aggregate annotations by counting entity mentions to generate feedback for training, thereby addressing the aforementioned limitations. We conduct experiments using various combinations of aggregate feedback and reward functions for comparison, focusing on Named Entity Recognition to validate our approach. The results suggest that sequence labeling can be learned from purely count-based labels, even at the sequence-level. Overall, this count-based method has the potential to significantly reduce annotation costs and variances, as counting entity mentions is more straightforward than determining exact boundaries.

## 1 Introduction

Sequence labeling represents a pervasive framework in Natural Language Processing (NLP), encompassing tasks such as Named Entity Recognition (NER), Part-Of-Speech tagging (POS), and Semantic Role labeling (SR), as well as Question Answering (QA) and Information Extraction (IE). These tasks have frequently been addressed using supervised learning approaches that require a labeled dataset with ground-truth sequences. Notable examples of supervised approaches for tackling sequence labeling include conventional Hidden Markov Models (HMM) (Kupiec, 1992), Conditional Random Fields (CRF) (Sha and Pereira, 2003), and (neural) sliding windows (Gallo et al., 2008), as well as deep neural networks such as Recurrent Neural Networks (RNN) (Graves, 2012) and, more recently, the Transformer architecture (Vaswani et al., 2017; Devlin et al., 2019). However, despite the continually improving performance in sequence labeling (Li et al., 2020; Zhang et al., 2023), supervised approaches are constrained by two technical limitations:

- **Training vs. evaluation:** There exists a common disparity between the training objective, typically a differentiable loss function, and the task-specific, possibly discrete evaluation metric, such as the F1-score. Consequently, minimizing the loss function might not directly optimize the evaluation measure, resulting in off-target training.
- **Labeling datasets:** In (standard) supervised machine learning, a labeled dataset D with fine-grained, task-specific ground-truth annotations *y*_1_, ..., *y*_*n*_ is normally required, but the process of acquiring such ground-truth annotations can be resource-intensive, depending on the application.

In reinforcement learning (RL), the learning progress is achieved by maximizing an arbitrary, possibly discrete reward-function *R* (the training objective). Since *R* is arbitrary, it exhibits two important properties: (a) *R* can directly represent and therefore optimize the evaluation measure (e.g. F1-score), and (b) training does not explicitly demand a labeled dataset D. Thus, RL naturally overcomes the above-mentioned limitations for supervised machine learning approaches (Keneshloo et al., 2020).

Yet, despite achieving notable success in gaming (OpenAI et al., 2019; Vinyals et al., 2019; Ye et al., 2020), robotics (Zhu et al., 2020; Akalin and Loutfi, 2021; Raffin et al., 2022), and planning (Zhu et al., 2021; Hamrick et al., 2021; Esteso et al., 2022), the utilization of reinforcement learning in natural language processing remains limited. While RL methods have been employed in, e.g., question answering (Choi et al., 2017; Buck et al., 2018) and information extraction (Narasimhan et al., 2016; Qin et al., 2018), the approaches considered are specifically engineered for rephrasing questions (Buck et al., 2018), denoising datasets (Qin et al., 2018), and assembling or condensing information (Choi et al., 2017; Narasimhan et al., 2016), as opposed to directly tackling the objective as an RL problem. As noteworthy exceptions, RL methods have been adopted to directly optimize policies in dialogue systems (Li et al., 2016; Lu et al., 2019; Liu et al., 2020) and paraphrase generation (Li et al., 2018; Qian et al., 2019; Siddique et al., 2020), including the fine-tuning processes of large language models such as InstructGPT (Ouyang et al., 2022) and GPT-4 (OpenAI, 2023), albeit with supervised pre-training.

Similarly, various methods have been proposed to address NER with RL (Wang et al., 2018; Yang et al., 2018; Wan et al., 2020; Peng et al., 2021a,b), but the proposed techniques only formulate secondary operations from an RL perspective. In some works, the RL methods are employed to pre-process incomplete or inaccurate annotations to accommodate strong(er) supervision by detecting and removing, sampling or cleaning negative and noisy instances (Yang et al., 2018; Peng et al., 2021a,b). In other works, the RL methods are instead utilized to complement a (traditional) supervised tagging approach by identifying and correcting invalid predictions (Wang et al., 2018; Wan et al., 2020).

The limited adoption of RL in NLP could, depending on the application (Uc-Cetina et al., 2022), be explained by the challenge of expressing the environment as an appropriate and well-defined sequential Markov decision process, as well as the notorious instability in training and low sample-efficiency when addressing complex learning problems or environments (Yu, 2018). In addition, designing a suitable reward function for effective learning can be challenging and is oftentimes accompanied by *delayed rewards* (Eick, 1988) or *sparse rewards* (Minsky, 1961), resulting in the well-known (temporal) *credit assignment problem* (Minsky, 1961). To mitigate this, meticulous *reward shaping* (Eschmann, 2021) or extensive *exploration* (Amin et al., 2021) may be necessary.

In this work, we present a novel RL-based approach that (a) considers sequence labeling exclusively from an RL perspective, and (b) does not strictly require token-level annotations for training. To accomplish this, we condense (or *aggregate*) standard token-level labels to summarize the ground-truth annotations by counting entity mentions. Then, we generate feedback for training by comparing the predicted and annotated entity counts. We experiment with combinations of feedback aggregation (i.e., multiple predictions are assigned a single reward signal) and reward functions, both count-based and *standard* (that is, with direct access to token-level labels), while evaluating our approach on the NER datasets CoNLL-2003 (Sang and Meulder, 2003), OntoNotes 5.0 (Hovy et al., 2006), and BC5CDR (Wei et al., 2016). In multiple instances for standard feedback, we obtain results that are competitive with a standard supervised baseline (i.e., that minimizes the cross-entropy loss), even outperforming the baseline by 2.33 points in F1-score on BC5CDR. For count-based feedback at the *sequence*-level, we obtain results that are only 11.37 and 9.56 points behind the standard baseline for CoNLL-2003 and BC5CDR, respectively. In summary, our findings indicate that learning sequence labeling tasks, such as NER, simply by counting entity mentions is possible and feasible, achieving remarkably solid performance. Such count-based methods could significantly reduce annotation costs as well as variances between annotations, as counting specific entity mentions is more straightforward and less subjective than determining precise entity boundaries.

## 2 Method

### 2.1 Preliminaries

(Deep) reinforcement learning algorithms are conventionally implemented through a sequential Markov decision process (MDP)—a mathematical framework used to determine a suitable environment *E* to be interacted with – and is denoted by M=(S,A,T,R) with state space S, action space A, transition function *T* (potentially stochastic) and reward function *R*. Subsequently, an agent (i.e, the learning system), whose actions $a_{t}\in A$ on states $s_{t}\in S$ are dictated by a (typically stochastic) policy function π(*s*_*t*_), interacts with the environment *E* over a sequence of discrete time-steps via state-action pairs (*s*_0_, *a*_0_), (*s*_1_, *a*_1_), ..., (*s*_*t*_, *a*_*t*_), and, in turn, observes rewards *r*_*t*_ = *R*(*s*_*t*_, *a*_*t*_, *s*_*t*+1_) upon each transition *s*_*t*+1_~*T*(·|*s*_*t*_, *a*_*t*_) as feedback from the environment. Ultimately, the objective function to be optimized by the policy function π is the expected cumulative discounted reward ${\text{E}}_{\pi }\left[\underset{t=0}{\sum }\gamma^{t}r_{t}\right]$ with a discount factor γ, when following the policy function π, i.e., by selecting the action *a*_*t*_~π(·|*s*_*t*_) that maximizes the expected cumulative discounted reward $R_{t}={\text{E}}_{\pi }\left[\underset{i=t}{\sum }\gamma^{i-t}r_{i}\right]$ at each time-step *t*.

### 2.2 Framework

We begin by formalizing the well-known framework of sequence labeling as a straightforward Markov decision process. Let (x,y)∈D denote a sequence of tokens *x* = *x*_1_, ..., *x*_*n*_ with ground-truth annotations *y* = *y*_1_, ..., *y*_*n*_ from a labeled dataset D (e.g., CoNLL-2003). We comprehend the sequence *x* = *x*_1_, ..., *x*_*n*_ with respective predictions ŷ = ŷ_1_, ..., ŷ_*n*_ (i.e., the actions *a*_1_, ..., *a*_*n*_ chosen by the agent) as an episode, and therefore construct the state space S from the starting state space $S_{0}$, intermediate state space $S_{I}$, and terminal state space $S_{T}$ as $S:=S_{\text{0}}\cup S_{\text{I}}\cup S_{\text{T}}$, with:


(1) $$\begin{array}{ll} S_{\text{0}} & :=\underset{x\in D}{⋃}\left\{\left(x,1\right)\right\} \end{array}$$

(2) $$\begin{array}{ll} S_{\text{I}} & :=\underset{x\in D}{⋃}\left\{\left(x,2\right),...,\left(x,|x|\right)\right\} \end{array}$$

(3) $$\begin{array}{ll} S_{\text{T}} & :=\underset{x\in D}{⋃}\left\{\left(x,|x|+1\right)\right\} \end{array}$$

where the states (x,t)∈S denote that position *t* (token *x*_*t*_) in sequence *x* shall be processed next.

Notice that, by construction of the state space S, an inherent disregard (or independence) for previously generated predictions ŷ_1_, ..., ŷ_*t*−1_ and subsequent predictions ŷ_*t*+1_, ..., ŷ_*n*_, respectively, is entailed, therefore suggesting (the application of) a memoryless policy function π. As further implied by the state space S, once an action a∈A is selected, each non-terminal state *s*_*t*_ = (*x, t*) is deterministically transformed into an intermediate (or terminal) state *s*_*t*+1_ = (*x, t*+1) by the transition function *T*, regardless of the assigned prediction, i.e., *T* describes a bijection from $S\backslash S_{\text{T}}$ to $S\backslash S_{\text{I}}$. The token labels task-specific to named entity recognition (e.g., O, B-PER, and I-MISC) must, of course, be accordingly represented by the action space A, and shall be characterized by non-negative integers 1,...,|A|. Lastly, we implement a framework of reward functions *R* to evaluate a sequence of consecutive predictions ŷ_*i*_, ..., ŷ_*j*_ (i.e., actions *a*_*i*_, ..., *a*_*j*_) against the ground-truth annotations *y*_*i*_, ..., *y*_*j*_, permitting any evaluation measure, and subsequently communicate the aggregated reward (or *feedback*) through the environment *E*.

### 2.3 Agent

We proceed by describing the architecture and behavior of our learning system (i.e. the agent) when operated by the policy function π. In value-based RL, such as Q-Learning, we choose some action a∈A based on the estimated state-action value *Q*_π_(*s*_*t*_, *a*) given state $s_{t}\in S\backslash S_{\text{T}}$ at time-step *t*. Specifically, this Q-value estimate represents the expected, long-term cumulative discounted reward **E**_π_[*R*_*t*_] when choosing action *a* at time-step *t* while being in state *s*_*t*_, and greedily following π thereafter. Thus, we estimate the state-action values *Q*(*s*_*t*_, ·), where *s*_*t*_ = (*x, t*) and *x* = *x*_1_, ..., *x*_*n*_, as follows:


(4) $$\begin{array}{ll} h_{1},...,h_{n} & =\text{Encoder}\left(x_{1},...,x_{n}\right) \end{array}$$

(5) $$\begin{array}{ll} {\hat{q}}_{t} & =Wh_{t}+b \end{array}$$

The Encoder is assumed to generate the contextualized representations (or *hidden states*) $h_{1},...,h_{n}\in {\mathbb{R}}^{d}$, with *d*∈ℕ, corresponding to the sequence *x*_1_, ..., *x*_*n*_, and the weight matrix $W\in {\mathbb{R}}^{|A|\times d}$ and bias term $b\in {\mathbb{R}}^{\left|A\right|}$ generate the state-action value predictions ${\hat{q}}_{1},...,{\hat{q}}_{n}\in {\mathbb{R}}^{\left|A\right|}$ from *h*_1_, ..., *h*_*n*_.

To address the dilemma of balancing *exploration* and *exploitation* (thereby defining our policy function π), we simply pursue an ϵ-greedy strategy, due to the relatively compact action-space A. Therefore, π(*s*) can be expressed as:


(6) $$\begin{array}{l} \pi \left(s\right)=\left\{ \begin{array}{cc} a\sim \text{Uniform}\left(A\right) & \text{with prob.}\epsilon \\ {arg max}_{a}Q\left(s,a\right) & \text{otherwise} \end{array}\right. \end{array}$$

where a~Uniform(A) denotes uniform sampling from A.

### 2.4 Reward schemes

We continue by establishing the partitioning mechanism by which the reward signals are delayed and aggregated. Each episode (i.e., a sample to be labeled) is segmented into independent subsections, each of which, once traversed and processed by the learning algorithm, is evaluated with an aggregated (and singular) reward signal. We segment an episode according to (a) the respective ground-truth annotations, and (b) the currently active *reward scheme*, which governs the *breadth* of a segment and, as a consequence, directly controls the degree by which the reward signals are delayed and aggregated. We implement the following reward schemes:

- **By action**: each prediction ŷ_1_, ..., ŷ_*n*_ is evaluated separately by *R*(*y*_*t*_, ŷ_*t*_).
- **By region**: a sequence of predictions ŷ_*i*_, ..., ŷ_*j*_ corresponding to a homogeneous (and maximal) sub-sequence of annotations *y*_*i*_, ..., *y*_*j*_ (i.e., named entities and non-entities) is evaluated in aggregate by *R*(*y*_*i*...*j*_; ŷ_*i*...*j*_).
- **By entity**: a sequence of predictions ŷ_*i*_, ..., ŷ_*j*_ generated by separating the annotations *y*_1_, ..., *y*_*n*_ behind each named-entity is evaluated in aggregate by *R*(*y*_*i*...*j*_, ŷ_*i*...*j*_).
- **k-grouped**: The concatenation of *k* completed sequences of predictions ŷ^1^, ..., ŷ^*k*^ with corresponding annotations *y*^1^, ..., *y*^*k*^ is evaluated in aggregate by *R*(*y*^1...*k*^, ŷ^1...*k*^), whereby the frequency of reward-signals decreases from *rewards-per-sample* to *samples-per-reward*.

**Figure 1:**
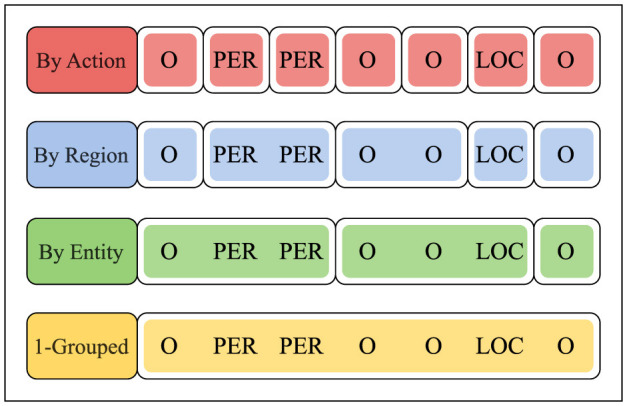
The considered reward schemes. Here, visually clustered sections (i.e., partitioned annotations) are to be evaluated in aggregate.

We provide an example for the *By-Action, By-Region, By-Entity*, and 1*-Grouped* reward scheme in Figure 1. The example assumes a gold-label sequence *y*_1_, ..., *y*_7_ with one LOC-type entity and one PER-type entity, spanning one LOC and two PER token labels, respectively. Obviously, the underlying input sequence *x*_1_, ..., *x*_7_ is irrelevant when calculating the rewards. For predictions ŷ_1_, ..., ŷ_7_, the *By-Action* scheme evaluates each individual prediction ŷ_*t*_ via the corresponding token label *y*_*t*_ through *R*. The *By-Region* scheme, in contrast, aggregates successive token labels into sub-sequences based on label class, e.g., consecutive LOC, O, and PER token labels in Figure 1. For predictions ŷ_*i*_, ..., ŷ_*j*_ over any such homogeneous sub-sequence, the feedback (one reward per section) is calculated in aggregate via the corresponding token labels *y*_*i*_, ..., *y*_*j*_ through *R*. As an extension, the *By-Entity* reward scheme combines two adjacent sections (or *regions*) as outlined in *By-Region* – e.g., by merging the initial O-region with the subsequent PER-region in Figure 1—thus providing one reward per two regions. Lastly, the *k-Grouped* reward scheme evaluates *k* prediction sequences ŷ^1^, ..., ŷ^*k*^ (using *k* = 1 in Figure 1) via the corresponding gold-label sequences *y*^1^, ..., *y*^*k*^, thus communicating one reward per *k* input sequences *x*^1^, ..., *x*^*k*^.

We simplify our notation by assuming that, supposing a sequence of predictions ŷ_*i*_, ..., ŷ_*j*_ to be evaluated in aggregate, the environment *E* communicates a reward signal *r*_*t*_ following each interaction *a*_*t*_ such that *r*_*i*_, ..., *r*_*j*−1_ are valueless (e.g., ⊥) and *r*_*j*_ represents the evaluation of the predictions ŷ_*i*_, ..., ŷ_*j*_ against the ground-truth labels *y*_*i*_, ..., *y*_*j*_. By design, the aggregated reward schemes are both *delayed* and *sparse*, because a singular non-empty reward (that is, our feedback for training) is only communicated through the environment *E* once a partition, as dictated by the current scheme, has been processed by the agent.

### 2.5 Reward functions

**Figure 2:**
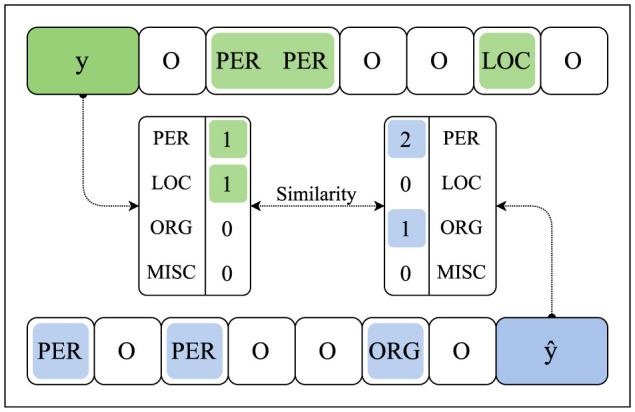
The method for calculating similarities by comparing the overall number of entities annotated in *y* (in green) and predicted in ŷ (in blue).

We consider various types of reward functions in our framework. Beyond Exact Match, Accuracy, and F1-score as a reward function *R*, we further experiment with several variants of the Cosine Similarity to compute a similarity between the predicted and target sequence. Apart from measuring the Cosine Similarity between the ground-truth annotations *y*_*i*_, ..., *y*_*j*_ (again, represented by non-negative integers 1,...,|A|) and the generated predictions ŷ_*i*_, ..., ŷ_*j*_, we further compare (i.e., calculate the similarity) of the entity counts per class between the predictions and ground-truth labels. This aggregate reward abstracts from the actual token-level annotations as, in contrast to standard reward functions such as Accuracy and Exact Match, these count-based reward functions only consider the *amount* of entities annotated in *y*_*i*_, ..., *y*_*j*_ and predicted in ŷ_*i*_, ..., ŷ_*j*_, see Figure 2.

Note that, when calculating count-based feedback, the function *R* is actually computed over vectors of entity counts count(*y*_*i*...*j*_) and count(ŷ_*i*...*j*_) rather than token labels *y*_*i*...*j*_ and predictions ŷ_*i*...*j*_ directly. In practice, count(*y*_*i*...*j*_) would, of course, be obtained from *x* via annotation. To simplify our notation, we assume that *R* handles the counting whenever necessary. For further details, see Section 4.1.

A cardinal problem with employing Cosine Similarity as a reward function *R*, however, becomes apparent when the generated predictions ŷ_*i*_, ..., ŷ_*j*_ (or amount of inferred entities) represent a multiple of the ground-truth annotations *y*_*i*_, ..., *y*_*j*_, because only the *directions* of vectors *A* and *B* are considered. As a consequence, a sequence of sub-optimal predictions ŷ_*i*_, ..., ŷ_*j*_ might be recognized as an optimal solution, as *R*(*y*, ŷ) = *R*(*y, y*). To address this problem, we incorporate a modification to the original formula to account for deviations in magnitude by which, therefore, a perfect reward is only achieved, if and only if *A* = *B*:


(7) $$\begin{array}{l} \sigma \left(A,B\right)=\frac{A\cdot B}{∥A∥\cdot ∥B∥}\cdot \left(1-\frac{\left|∥A∥-∥B∥\right|}{∥A∥+∥B∥}\right) \end{array}$$

### 2.6 Algorithm

We outline our single-episode learning procedure in Algorithm 1 where, because our reward schemes are considered a characteristic of the environment *E* (to simplify our notation), the reward schemes and functions are indirectly addressed via *E*(*a*_*t*_|*R*). In Line 11, the model parameters θ are optimized according to the Mean Squared Error between the predicted and resulting state-action values ${\hat{q}}_{1},...,{\hat{q}}_{n}$ and *q*_1_, ..., *q*_*n*_, respectively:


(8) $$\begin{array}{l} L\left(\theta \right)=\frac{1}{n}\overset{n}{\underset{t=1}{\sum }}{\left(q_{t}-{\hat{q}}_{t}\right)}^{2} \end{array}$$

**Algorithm 1:**
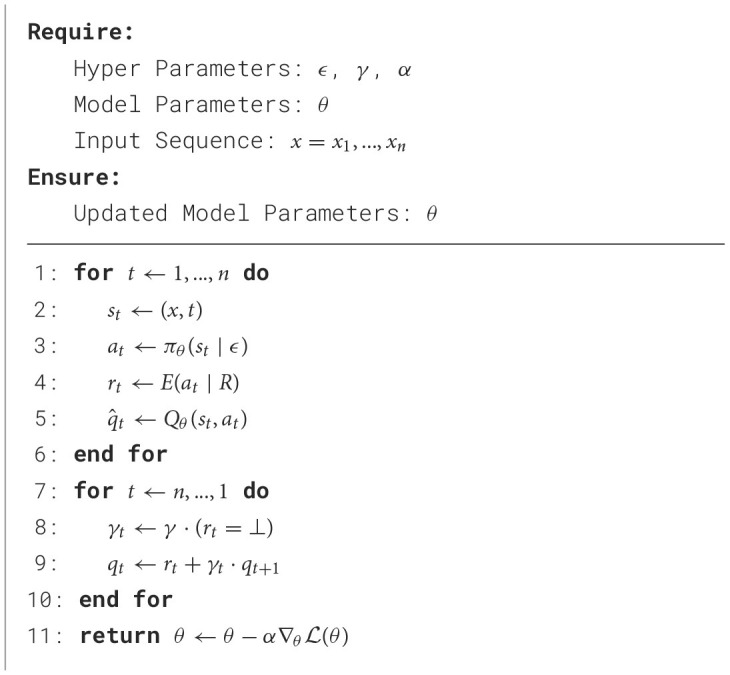
Update step.

We implemented several modifications to standard Deep Q-Learning (Mnih et al., 2013):

Firstly, we eliminated the *experience replay* (Lin, 1992; Fedus et al., 2020), because a sequence of continuous predictions ŷ_*i*_, ..., ŷ_*j*_ (as determined by the reward scheme) might, in consequence, not be evaluated in aggregate, since the evaluation of a particular prediction ŷ_*t*_ is dependent on the evaluation of the associated sequence ŷ_*i*_, ..., ŷ_*j*_ containing ŷ_*t*_. Additionally, by discarding the experience mechanism, each prediction ŷ_1_, ..., ŷ_*n*_ can be computed from the same contextualized representation *h*_1_, ..., *h*_*n*_, requiring only a single encoding per sequence *x*_1_, ..., *x*_*n*_, as the parameters θ are yet to be updated.

Secondly, because the aggregated subsections are evaluated separately (predictions are independent by design of our framework), we introduce *gated discounting* (via γ_*t*_) to encourage short-term strategies within aggregated subsections and discourage long-term strategies across aggregated subsections. To accomplish this, we condition γ on the received feedback:


(9) $$\begin{array}{l} \gamma_{t}:=\left\{ \begin{array}{cc} 0 & \text{if }r_{t}\neq ⊥\\ \gamma & \text{otherwise} \end{array}\right. \end{array}$$

Note that γ_*t*_ is always 0 whenever a non-empty reward-signal is observed by the learning algorithm, effectively separating two consecutive subsections (as seen by the agent).

Thirdly, we replace the original *Q*-value estimates (Mnih et al., 2013) with non-terminal ground-truth *Q*-values *q*_*t*_ to propagate the upcoming, non-empty reward-signals directly within their respective partitions.


(10) $$\begin{array}{ll} q_{t} & =r_{t}+\gamma_{t}\cdot q_{t+1} \end{array}$$

By introducing this modification, we associate a single (yet discounted) evaluation *r*_*j*_ with a complete sequence of predictions ŷ_*i*_, ..., ŷ_*j*_ and, as opposed to producing purely local estimates, encourage the agent to estimate the sectional evaluation of ŷ_*i*_, ..., ŷ_*j*_ through each *Q*-value estimate ${\hat{q}}_{i},...,{\hat{q}}_{j}$.

### 2.7 Experiments

We utilize a comparatively lightweight BERT checkpoint (bert-base-cased^1^) sourced from HuggingFace as our base-model. This checkpoint is configured with 12 transformer blocks, a hidden dimension of 768, and 12 attention heads, totaling approximately 110 million pre-trained parameters. As a consequence, the output-layer *W* (which is used for classification) is composed of 768×|A| parameters, which we randomly initialize from $U\left(-\sqrt{k},\sqrt{k}\right)$, where $k=\frac{1}{768}$.

The individual experiments are conducted over 400 rounds, during each of which 250 updates are performed on the model-parameters θ, amounting to 100,000 updates per experiment. The updates are performed over batches of 8 sequences, sampled uniformly at random. The exploration-exploitation dilemma is addressed by selecting ϵ = max (0.005, 0.5^*round*−1^), such that ϵ is never below 0.5%, while discounting is handled with γ = 1. We maintain a constant learning rate α of 1e-5 and utilize AdamW with standard parameters for optimization. We calculate the learning system's performance using *seqeval*,^2^ an open-source framework for sequence labeling evaluation.

**Figure 3:**
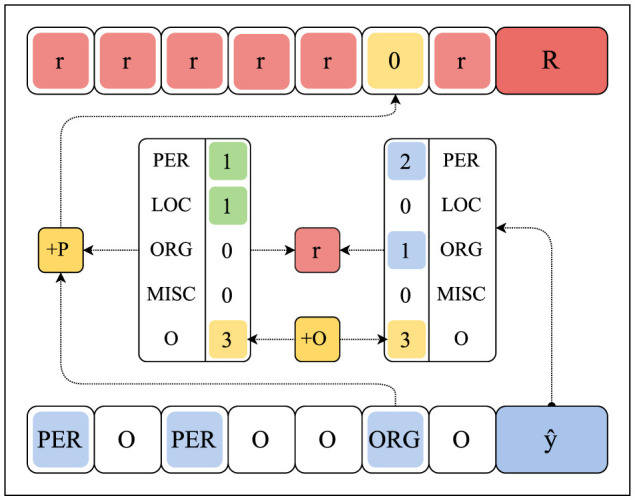
The method by which +O and +P counting variants (in yellow) calculate and provide feedback. In this sketch, the reward signal *r* (in red) is communicated at the sequence-level 1*-Grouped*. The +O counting variant includes contiguous O-intervals. The +P counting variant provides 0-rewards for predictions *y*_*t*_ (in blue) that are impossible given the ground-truth annotations *y* (in green).

We implement Exact Match, Accuracy, F1-score, and the enhanced Cosine Similarity from Equation 7 as standard reward functions. Additionally, we use the enhanced Cosine Similarity function for comparing the entity counts contained in the ground truth annotations and predictions, utilizing four configurations: Counts, Counts +O, Counts +P, and Counts +OP. The Counts function enumerates all annotated or predicted named entities, while +O variants also consider the contiguous O-intervals (regions of consecutive O token labels) in a sequence, and +P variants may only enumerate the true-positive entity counts (and O-intervals, if +O), directly assigning a 0-reward to predictions ŷ_*t*_ that are *impossible* considering the annotated entity counts, see Figure 3. We consider the following reward schemes: *By-Action, By-Region, By-Entity*, 1*-Grouped*, 2*-Grouped*, and 4*-Grouped*.

We evaluate our approach on the following datasets for named entity recognition in English: CoNLL-2003, OntoNotes 5.0, and BC5CDR. CoNLL-2003 (Sang and Meulder, 2003), a dataset sourced from news articles, encompasses four categories of named entities: person (PER), location (LOC), organization (ORG), and miscellaneous (MISC). OntoNotes 5.0 (Hovy et al., 2006), compiled from news articles, weblogs, and dialogues, presents a wider array of named entities, featuring 18 categories that include 11 entity classes (such as building, event, and product) and 7 value types (such as percent, time, and quantity). The original BC5CDR dataset (Wei et al., 2016) consists of biomedical documents annotated for mentions of diseases and chemicals. However, for our purposes, we utilize the sentence-based version pre-processed for T-NER (Ushio and Camacho-Collados, 2021). We employ the default dataset splits (see Table 1).

**Table 1:** The number of instances (sequences) per default dataset split.

	**CoNLL-2003**	**OntoNotes 5.0**	**BC5CDR**
Train	14.041	59.924	5.228
Validation	3.250	8.528	5.330
Test	3.453	8.262	5.865
Total	20.744	76.714	16.423

Finally, to establish a benchmark for comparison, we introduce a standard baseline. This baseline is obtained by training our base-model (BERT) via standard supervised learning, minimizing the cross-entropy loss. In contrast to our RL approach, the baseline has direct access to the token-level annotations. The experimental procedures and evaluations are otherwise identical.

## 3 Results

We showcase our main experimental results in Table 2. The reported results are clustered by standard feedback, count-based feedback, and the standard baseline, and represent the maximum observed F1-scores on the validation split (averaged over 5 runs). In the following, we distinguish between standard and count-based feedback.

**Table 2:** The average maximum F1-scores (computed over validation datasets) with respective standard deviations.

	**CoNLL-2003**					
	***By-Action***	***By-Region***	***By-Entity***	*1**-Grouped***	*2**-Grouped***	*4**-Grouped***
Exact Match	**94.32** **±0.06**	93.81 ± 0.06	91.52 ± 1.01	4.38 ± 0.05	4.17 ± 0.33	4.42 ± 0.47
Accuracy	**94.32** **±0.06**	**93.91** **±0.10**	**93.25** **±0.20**	**87.43** **±2.13**	58.21 ± 5.29	47.69 ± 3.87
F1-Score	83.72 ± 2.72	92.23 ± 0.31	91.41 ± 0.61	84.94 ± 4.05	**76.36** **±2.10**	**61.16** **±11.23**
Cosine Sim.	93.20 ± 0.39	90.44 ± 0.65	87.21 ± 2.24	74.64 ± 0.96	68.11 ± 5.68	50.18 ± 4.42
Counts	**87.38** **±3.58**	75.78 ± 6.64	33.83 ± 13.10	9.59 ± 1.34	9.21 ± 3.86	7.99 ± 1.69
Counts +O	**87.38** **±3.58**	77.74 ± 8.48	**74.28** **±6.82**	**82.79** **±2.26**	74.53 ± 5.21	49.06 ± 20.78
Counts +P	**87.38** **±3.58**	74.76 ± 8.23	50.33 ± 4.84	8.95 ± 1.67	14.15 ± 8.14	26.33 ± 15.38
Counts +OP	**87.38** **±3.58**	**81.74** **±3.49**	72.61 ± 4.68	78.96 ± 3.87	**79.76** **±3.59**	**78.10** **±3.70**
Baseline	94.16 ± 0.08					
	**OntoNotes 5.0**					
	***By-Action***	***By-Region***	***By-Entity***	*1**-Grouped***	*2**-Grouped***	*4**-Grouped***
Exact Match	**86.10** **±0.06**	82.51 ± 0.56	57.35 ± 4.69	2.34 ± 0.11	2.49 ± 0.44	1.71 ± 0.01
Accuracy	**86.10** **±0.06**	**85.30** **±0.08**	**81.83** **±0.26**	24.04 ± 4.04	2.05 ± 1.39	2.18 ± 1.02
F1-Score	58.75 ± 5.49	81.86 ± 0.50	66.86 ± 5.62	**51.14** **±4.33**	**33.14** **±2.57**	**25.26** **±2.39**
Cosine Sim.	78.61 ± 0.34	55.37 ± 1.14	52.52 ± 2.64	28.20 ± 0.56	17.33 ± 0.88	16.68 ± 1.15
Counts	**56.42** **±3.84**	58.88 ± 5.38	11.12 ± 1.06	4.94 ± 0.41	6.53 ± 1.27	5.44 ± 0.80
Counts +O	**56.42** **±3.84**	59.05 ± 7.67	53.97 ± 6.18	53.31 ± 5.41	**57.85** **±2.18**	**48.61** **±5.35**
Counts +P	**56.42** **±3.84**	53.68 ± 10.50	11.72 ± 0.47	19.86 ± 18.51	44.84 ± 4.80	25.90 ± 13.86
Counts +OP	**56.42** **±3.84**	**62.35** **±9.45**	**56.32** **±8.13**	**55.93** **±1.33**	55.35 ± 2.71	45.12 ± 0.91
Baseline	86.06 ± 0.06					
	**BC5CDR**					
	***By-Action***	***By-Region***	***By-Entity***	*1**-Grouped***	*2**-Grouped***	*4**-Grouped***
Exact Match	**85.78** **±0.11**	84.91 ± 0.21	**84.53** **±0.22**	1.55 ± 0.58	4.54 ± 1.89	3.21 ± 1.84
Accuracy	**85.78** **±0.11**	**84.95** **±0.15**	84.45 ± 0.07	73.50 ± 1.76	68.68 ± 3.70	48.13 ± 5.40
F1-Score	85.00 ± 0.12	84.20 ± 0.20	82.88 ± 1.53	**81.79** **±0.20**	**81.31** **±0.37**	**55.39** **±7.74**
Cosine Sim.	84.03 ± 0.10	81.42 ± 0.04	77.70 ± 0.21	71.06 ± 1.70	64.62 ± 0.35	50.80 ± 3.61
Counts	**83.56** **±0.07**	77.99 ± 1.17	22.05 ± 8.15	17.24 ± 1.38	8.21 ± 4.70	5.72 ± 0.83
Counts +O	**83.56** **±0.07**	80.70 ± 0.48	68.02 ± 2.17	58.23 ± 5.55	39.51 ± 4.76	27.44 ± 11.59
Counts +P	**83.56** **±0.07**	78.28 ± 1.45	41.86 ± 0.43	30.99 ± 10.69	37.73 ± 7.98	24.68 ± 4.21
Counts +OP	**83.56** **±0.07**	**81.46** **±0.67**	**74.81** **±1.53**	**73.89** **±1.77**	**62.74** **±2.68**	**47.78** **±0.49**
Baseline	83.45 ± 0.40					

The highest obtained F1-scores for *standard* and *count-based* reward functions are highlighted in bold.

### 3.1 Standard feedback

Unsurprisingly, the results reached by training the learner using standard reward functions that calculate feedback based on token-level annotations are relatively consistent when combined with sub-sequence reward schemes like *By-Action, By-Region*, and *By-Entity*. For CoNLL-2003 and OntoNotes 5.0, the highest results (using Accuracy as feedback) are generally competitive with the standard supervised baseline of 94.16 and 86.06 points in F1-score, respectively. Notably, the highest results for BC5CDR outperform the standard baseline (83.45 F1-score) by 1.08 to 2.33 points in F1-score for reward schemes *By-Action* to *By-Entity*, even remaining competitive for feedback aggregated to sequence-level 2*-Grouped* (81.31 F1-score).

As we transition from reward scheme *By-Action* to sequence-level 1*-Grouped*, performance naturally deteriorates as feedback becomes more aggregated; this decrease is especially noticeable for OntoNotes 5.0, with the highest results falling by 30.69 points in F1-score, whereas the decrease for CoNLL-2003 and BC5CDR is limited to 5.82 and 2.74 points, respectively. Notably, when switching from 1*-Grouped* to 2*-Grouped*, the highest results are relatively stable for BC5CDR, only dropping by 0.48, while results for CoNLL-2003 and OntoNotes 5.0 decrease by 11.07 and 18.00 points in F1-score.

When providing feedback via the *By-Action* reward scheme, Exact Match and Accuracy as reward functions (whereby each individual prediction ŷ_*t*_ is assigned a 0/1-reward) produce the highest results across all evaluated scenarios and datasets, even outperforming the standard supervised baseline. Notice that when the feedback is conveyed as an F1-score, performance drops significantly. While the reward signals for *By-Action* are communicated as *reward-per-action*, the F1-score, unlike Exact Match or Accuracy, is generally not applicable to NER when calculated over singular token-labels. To illustrate this, we propose a scenario where the ground-truth label is *y*_*t*_ = I-PER and the prediction is ŷ_*t*_ = B-PER. In this case, the F1-score calculated by *seqeval* yields F1(*y*_*t*_, ŷ_*t*_) = 1.0, whereby the learner is unable to distinguish between B-PER and I-PER.

For aggregate feedback via *By-Region* and *By-Entity*, Accuracy mostly yields the highest results for all datasets, with performance shrinking by 0.66, 3.47, and 0.50 points for CoNLL-2003, OntoNotes 5.0, and BC5CDR, respectively. In comparison, feedback produced by the much less informative Exact Match achieves only slightly worse results for CoNLL-2003 and BC5CDR. However, results plummet by 24.48 points for OntoNotes 5.0. Once feedback is provided at sequence-level 1*-Grouped* to 2*-Grouped*, we observe a notable decrease in the results yielded by Accuracy and Exact Match. In general, F1-score achieves the highest results for feedback provided at sequence-level *k-Grouped*, remaining remarkably stable as *k* increases, with performance dropping by at most 23.78, 25.88, and 26.40 points for CoNLL-2003, OntoNotes 5.0, and BC5CDR.

### 3.2 Count-based feedback

In general, count-based feedback is expected to facilitate a reduced performance when compared to token-based supervision, as it provides less concrete feedback to the learning system. For instance, when considering the sub-sequence reward schemes *By-Action, By-Region*, and *By-Entity*, we observe a substantial decrease in performance between conventional and count-based reward functions.

*However*, when considering sequence-level reward aggregation 1*-Grouped*, the difference in performance between standard feedback (e.g., F1-score) and count-based feedback (e.g., Counts +OP) is surprisingly low. Specifically, metrics only decrease by 4.64 and 7.90 points for CoNLL-2003 and BC5CDR, and increase by 4.79 points for OntoNotes 5.0, when switching from F1-score to Counts +OP. This is remarkable given the stark contrast in training regimes and, even more so, the supposedly unreliable information conveyed by count-based feedback over feedback directly computed from token-level annotations. Furthermore, the results gained via Counts +O and Counts +OP are competitive with token-based feedback for sequence-level reward aggregation *k-Grouped*, even outperforming the strongest standard reward functions for CoNLL-2003 and OntoNotes 5.0 (i.e., F1-score and Accuracy) by 16.94 and 24.71 points. Obviously, the highest scores are generally obtained via *informed* counting with Counts +OP, as it provides the most nuanced feedback to the learning system, whereas naïve (or *uninformed*) counting, as executed in Counts, consistently yields diminished performance for all experiments.

In addition, results remain reasonably consistent across *By-Action* to 4*-Grouped* for count-based feedback, exhibiting a maximum difference (over highest scores) of 13.10, 13.74, and 35.78 points for CoNLL-2003, OntoNotes 5.0, and BC5CDR, respectively. In contrast, results for token-based feedback have relatively high variance, displaying a maximum difference (again, over highest scores) of 33.16, 60.84, and 60.52 for CoNLL-2003, OntoNotes 5.0, and BC5CDR.

Notice that, by design of the reward schemes *By-Region* and *By-Entity* (and, trivially, for *By-Action*), even when considering count-based annotations, the learner is implicitly provided with information about the underlying token-level annotations, as partitions from *By-Region* and *By-Entity* are constructed such that they comprise at most one named entity. The reward schemes *By-Region* and *By-Entity* thus provide an interesting perspective on the differences in performance when transitioning from *By-Region* to *By-Entity* to 1*-Grouped*. For instance, looking at the highest results for count-based feedback, we observe a significant decrease in performance from *By-Region* to *By-Entity*, suggesting that recognizing the boundaries of singular named entities is particularly challenging when provided only with count-based feedback. However, when feedback aggregation is elevated from *By-Entity* to 1*-Grouped* (i.e., to sequence-level), results decrease only slightly for OntoNotes 5.0 and BC5CDR, even increasing by 8.51 points for CoNLL-2003, indicating that detecting (the boundaries of) multiple named entities is relatively straightforward when viewed from *By-Entity*.

## 4 Discussion

The findings outlined in Section 3 demonstrate that learning sequence labeling tasks, such as NER, with aggregate feedback is feasible, even when the feedback is derived entirely by counting entity mentions per class, although with some obvious caveats. In comparison to feedback computed from token-level annotations, the count-based rewards facilitate a reduced learning capacity, providing relatively imprecise and, in part, unreliable information to the learning algorithm. By design, reward signals derived from entity counts over generated predictions ŷ_1_, ..., ŷ_*n*_ and ground-truth annotations *y*_1_, ..., *y*_*n*_ only communicate information pertaining the *existence* of an entity, not its respective *boundaries*. Nevertheless, overall results are remarkably solid considering these constraints.

In Section 1, we briefly explore the advantages of utilizing RL methods over standard supervised learning techniques, especially pertaining to the implications of an arbitrary reward function *R* dictating the learning progress. This function *R* can directly represent and therefore optimize the evaluation measure, including the F1-score. Looking at Table 2, the experiments on standard reward functions, which calculate feedback from token-level annotations, support this assumption for sequence-level feedback 1*-Grouped* (and beyond), as designing the function *R* to compute the current F1-score between the gold-labels *y*_1_, ..., *y*_*n*_ and the predictions ŷ_1_, ..., ŷ_*n*_) is indeed shown to outperform the token-based alternatives, such as Accuracy. However, while token-based feedback at the sequence-level *k-Grouped* achieves its greatest potential when representing the F1-score between *y*_1_, ..., *y*_*n*_ and ŷ_1_, ..., ŷ_*n*_, we observe that count-based feedback often surpasses token-based feedback (including the F1-score) while achieving more consistent performance.

As detailed in SubSection 3.2, our results reflect the significance of *informed counting*, as demonstrated by Counts +OP versus Counts. While Counts +O (considering contiguous O-intervals, i.e., non-entities) and Counts +P (providing 0-rewards on false-positive counts) both provide some contrasting information to the learning system, the resulting increase in performance from Counts +O overshadows the improvements gained from Counts +P. Furthermore, when integrated as Counts +OP, a significant and consistent improvement in performance (and standard deviation) is achieved over both configurations, especially for CoNLL-2003 and BC5CDR.

Notably, our results suggest that learning progress for count-based feedback may be negatively influenced by the number of entity types, that is, the cardinality of the action space A. For instance, even when considering the reward scheme *By-Action*, the difference in performance to the standard baseline is 6.78 points for CoNLL-2003 and -0.11 points for BC5CDR, which have |A|=9 and |A|=5, respectively. In comparison, this difference is exacerbated to 29.64 points for OntoNotes 5.0, where |A|=37, thus raising questions regarding the suitability of count-based feedback at the sequence-level when handling sequence labeling tasks with a relatively large action space A.

As an alternative explanation, the divergence in performance could instead be caused by class label imbalances during training. In fact, OntoNotes 5.0 (*k* = 18 classes) exhibits the greatest label imbalance, with 4 entity types constituting more than 66% of overall entity mentions, whereas CoNLL-2003 (*k* = 4) and BC5CDR (*k* = 2) feature a more balanced class distribution, see Table 3. Hence, our overall results might be improved by employing a more sophisticated algorithm for sampling training instances from D (as opposed to uniform random sampling). We provide further material for this correlation in Section 4.1.

**Table 3:** The absolute (*c*_*i*_) and relative (*c*_*i*_/*n*) entity counts per label class and dataset (train split).

	**CoNLL-2003**			**OntoNotes 5.0**			**BC5CDR**	
	**(***k* = 4**)**			**(***k* = 18**)**			**(***k* = 2**)**	
	*c*_*i*_	*c*_*i*_/*n*		*c*_*i*_	*c*_*i*_/*n*		*c*_*i*_	*c*_*i*_/*n*
LOC	7,140	30.38%	PER	15,429	18.86%	CHEM	5,203	55.43%
PER	6,600	28.09%	GPE	15,405	18.83%	DISE	4,182	44.56%
ORG	6,321	26.90%	ORG	12,820	15.67%	–	–	–
MISC	3,438	14.63%	DATE	10,922	13.48%	–	–	–
–	–	–	(...)	27,252	33.30%	–	–	–
Total	23,499	100%	Total	81,828	100%	Total	9,385	100%

### 4.1 Case study

In this Section, we provide some concrete model predictions to demonstrate how count-based feedback is calculated and distributed over predictions, thus promoting a more comprehensive understanding of our methodology. In addition, considering the imprecision and granularity of count-based feedback, where no boundary-related information is communicated, the exemplary predictions shall emphasize the remarkable performance on boundary detection.

The following examples were generated for CoNLL-2003 and obtained by training with count-based feedback (Count +OP) at the sequence-level 1*-Grouped* as described in Section 2.7. The example predictions and resulting per-class and absolute model performance are presented in Tables 4, 5, respectively. Notably, although no token-level information is communicated via count-based feedback, the learner reaches an astonishing performance on boundary detection, as demonstrated by examples (a) through (f) in Table 4.

**Table 4:** A selection of predictions (and common mistakes) from a model trained on CoNLL-2003 via Counts +OP with sequence-level feedback 1-*Grouped*.

	CoNLL-2003		
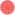 PER 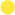 MISC 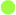 LOC 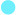 ORG		
(a)	**Labels**:	Interfax said Judge Olga Lavrentyeva ordered the confiscation of several overcoats, suits and shirts which vendor Valery Ivankov was illegally trading on Moscow streets.
	**Model**:	Interfax said Judge Olga Lavrentyeva ordered the confiscation of several overcoats, suits and shirts which vendor Valery Ivankov was illegally trading on Moscow streets.
(b)	**Labels**:	In Milwaukee, Marc Newfield homered off Jose Parra (5-4) leading off the bottom of the 12th as the Brewers rallied for a 5-4 victory over the Minnesota Twins.
	**Model**:	In Milwaukee, Marc Newfield homered off Jose Parra (5-4) leading off the bottom of the 12th as the Brewers rallied for a 5-4 victory over the Minnesota Twins.
(c)	**Model**:	Coach Berti Vogts has called up a virtually identical squad for next week's friendly against Poland – Germany's first match since Euro 96.
	**Model**:	Coach Berti Vogts has called up a virtually identical squad for next week's friendly against Poland – Germany's first match since Euro 96.
(d)	**Model**:	Prime Minister John Major says the 300-year-old union of the Scottish and English parliaments will be a main plank in his Conservative Party's election platform.
	**Model**:	Prime Minister John Major says the 300-year-old union of the Scottish and English parliaments will be a main plank in his Conservative Party's election platform.
(e)	**Model**:	The move to Bergamo-based Atalanta reunites Lentini, who fell out with ex-Milan coach Fabio Capello last season, with his former coach at Torino, Emiliano Mondonico.
	**Model**:	The move to Bergamo-based Atalanta reunites Lentini, who fell out with ex-Milan coach Fabio Capello last season, with his former coach at Torino, Emiliano Mondonico.
(f)	**Model**:	“I request the immediate repatriation of Kim In-so to North Korea,” North Korean Red Cross president Li Song-ho said to his southern counterpart, Kang Young-hoon.
	**Model**:	“I request the immediate repatriation of Kim In-so to North Korea,” North Korean Red Cross president Li Song-ho said to his southern counterpart, Kang Young-hoon.

The examples (a) and (f) were pruned for brevity.

**Table 5:** The overall metrics achieved by training on CoNLL-2003 via Counts +OP with sequence-level feedback 1*-Grouped*.

	**CoNLL-2003**				
	*c*_*i*_	**Precision**	**Recall**	**F1-Score**	**Accuracy**
PER	1,842	90.26	93.54	91.87	–
LOC	1,837	71.20	79.26	75.01	–
ORG	1,341	72.52	79.12	75.68	–
MISC	922	44.58	45.99	45.27	–
Overall	5,942	73.24	78.49	75.78	88.81

The absolute entity mentions per label class and dataset (validation split) are denoted by *c*_*i*_.

We observe that incorrect label predictions are almost always encountered in one of the following cases: scenario (1), wherein the learning system (entirely) ignores MISC- and ORG-class tokens, as illustrated by examples (c) and (d), or scenario (2), wherein the learning system only detects the LOC-related segment in MISC- and ORG-class entities, as illustrated by examples (e) and (f). Note that scenario (1) can result from scenario (2). To support this observation, we present the resulting confusion matrix (per token) in Table 6. Additionally, as anticipated in Section 4, we observe an apparent decrease in performance for infrequent token labels, specifically the previously mentioned MISC-class entities, see Tables 5, 6. However, looking more closely at Tables 3, 5, this correlation is only partially supported. The results indicate that the overall PER and MISC-class mentions are roughly proportional to the corresponding F1-Scores, yielding PER-to-MISC ratios of 1.92 (mentions) and 2.03 (F1-Score). In contrast, despite the notably more frequent mentions of LOC over ORG-class entities (7140 vs. 6321 mentions, ratio 1.13), we observe an unexpected, marginal decrease in performance (75.01 vs. 75.68 F1-Score, ratio 0.99). A similar pattern can be observed when comparing LOC with PER-class entities, having ratios of 1.08 (mentions) and 0.82 (F1-Score). Overall, these results suggest that absolute mention frequency does not consistently correspond with performance.

**Table 6:** The confusion matrix obtained by training on CoNLL-2003 via Counts +OP with sequence-level feedback 1*-Grouped*.

		**Prediction**				
		**O**	**PER**	**LOC**	**ORG**	**MISC**
Gold Label	O	0.990	0.001	0.006	0.002	0.001
	PER	0.008	0.979	0.004	0.008	0.001
	LOC	0.016	0.003	0.939	0.038	0.004
	ORG	0.112	0.008	0.048	0.827	0.005
	MISC	0.265	0.019	0.097	0.060	0.560

The confusion scores are normalized per gold label. The colors scale with the scores in the matrix. Higher scores mean higher intensity.

#### 4.1.1 Reward calculation

To demonstrate the procedure for calculating feedback as illustrated in Figure 3, we manually determine the count-based feedback under reward scheme 1*-Grouped* for examples (b) and (e) in Table 4. Let count_*c*_(*y*) denote the overall entity counts for class *c* in a sequence *y*. For CoNLL-2003, we further denote by count(*y*) the ordered sequence of (entity) counts count_*c*_(*y*) for *c*=PER, LOC, ORG, MISC, and O (whenever +O counting variants are considered). We obtain the following ordered sequences for examples (b) and (e):


$$\begin{array}{l} \text{b) count}\left(y\right)=\left[2\;1\;2\;0\;6\right]\text{and}\\ \;\text{count}\left(ŷ\right)=\left[2\;1\;2\;0\;6\right]\\ \text{e) count}\left(y\right)=\left[3\;0\;2\;2\;7\right]\text{and}\\ \;\text{count}\left(ŷ\right)=\left[3\;2\;2\;0\;8\right] \end{array}$$

where the values 6, 7, and 8 indicate the number of contiguous O-intervals (beware the punctuation) for the respective gold-label sequence *y* and predictions ŷ. Subsequently, we calculate the modified cosine similarity σ between count(*y*) and count(ŷ) to obtain our (global) reward signal *r* over the predictions ŷ for the learning system (see Equation 7):


$$\begin{array}{l} \text{b)}\text{reward }r=\sigma \left(\left[2\;1\;2\;0\;6\right],\left[2\;1\;2\;0\;6\right]\right)\\ \;=1.00\left(1.00\text{ for +O}\right)\\ \text{e)}\text{reward }r=\sigma \left(\left[3\;0\;2\;2\;7\right],\left[3\;2\;2\;0\;8\right]\right)\\ \;\approx 0.76\left(0.89\text{ for +O}\right) \end{array}$$

Further, when +P counting variants are considered, we assign a (local) 0-reward to predictions ŷ_*t*_ that are impossible given the ground-truth counts. For instance, in example (e), we observe that count_LOC_(*y*) = 0 and count_LOC_(ŷ) = 2, thus resulting in 0-rewards for any prediction ŷ_*t*_ that matches the label class LOC.

**Note:** In this work, we compute singular rewards (per partition) based on overall entity counts, jointly. One could, however, provide one reward signal per entity class instead, e.g., by computing and evaluating the deviation between count_*c*_(*y*) and count_*c*_(ŷ) directly, thus assigning feedback at the sub-sequence level without requiring token-level annotations. This modification would naturally extend and generalize the +P counting variants.

### 4.2 Related work

As suggested in Section 1 and discussed in Section 4, reinforcement learning techniques can—by virtue of an arbitrary reward function *R*—potentially overcome the aforementioned limitations of (standard) supervised machine learning, namely the prevalent mismatch between the training objective and the evaluation measure, as well as the requirement of a labeled dataset D with fine-grained annotations (e.g., at token-level).

Yet, despite this apparent potential, and although various RL methods have been proposed to *complement* sequence labeling approaches for weakly supervised learning—where training is conducted on approximate annotations, meaning *incomplete, inexact*, or *inaccurate* (Zhou, 2018), as fine-grained, high-quality annotations are generally expensive to assemble—we notice that RL techniques are generally not utilized to (a) address NLP tasks *directly*, that is, without extending or requiring a pre-trained model, and (b) overcome the aforelisted limitations for supervised machine learning (in NLP), particularly the reliance on fine-grained annotations.

For instance, Yang et al. (2018) propose an approach that involves *partial annotation* learning to address the incomplete annotations, followed by an RL-based instance selector that identifies positive (or *clean*) samples for training, thus handling the inaccurate annotations. In similar fashion, Peng et al. (2021a,b) propose an RL-based instance selector for pre-training a classifier, followed by (a) training on negative samples (Peng et al., 2021a), or (b) an adversarial training mechanism (Peng et al., 2021b) to improve the classifier's robustness against incomplete or inaccurate annotations. In contrast, Wang et al. (2018) and Wan et al. (2020) employ an RL-based system for detecting and rectifying (a) incorrect predictions generated by some pre-trained tagging system (Wang et al., 2018), or (b) incorrect token-labels from annotations auto-generated via distant supervision (Wan et al., 2020).

In this work, we have thus introduced (a) a framework for sequence labeling that directly addresses the problem from a standalone and value-based RL perspective, without requiring a pre-trained model, and (b) the utilization of count-based rewards for training that are obtained by counting entity mentions at the sequence-level (as opposed to considering token-level annotations).

Notably, token-label counts have previously been leveraged to formulate a consistency loss function to maintain consistent entity mentions across paraphrased sequences (Chen et al., 2020). Beyond this, we are not aware of comparable count-based approaches in NLP. However, count-based learning has been investigated in various computer vision settings, such as Weakly Supervised Object Detection (Hsu and Li, 2020), where object-counts are considered over ground-truth candidate proposals (e.g., object classes and specific locations), and Crowd Counting (Savner and Kanhangad, 2023), where count-based annotations are utilized instead of point-level annotations. In other works, a clustering framework for Multiple Instance Learning is presented (Oner et al., 2020), where the training approach relies solely on collection-level annotations that indicate the number of distinct classes within a collection of instances, labeled *unique class counts*. Count-based learning has also been employed for weakly-supervised temporal localization (Schroeter et al., 2019), specifically the localization and detection of instantaneous event occurrences (lasting for one time-step) in sequential data, with training being conducted on occurrence-counts only. Unfortunately, when transferred to NER, this method requires token-level annotations, since the problem definition assumes that event occurrences (named entities) are instantaneous (composed of a single token).

## 5 Conclusion and future work

In this work, we presented a unique method to sequence labeling that leverages count-based annotations, e.g., obtained by counting (rather than marking) specific entity mentions in a text, for training. Therefore, we introduced a framework that directly formulates the sequence labeling task from an RL perspective. To validate our approach for NER, we experimented with various degrees of feedback aggregation (multiple predictions are assigned a single reward) in combination with *standard* and *count-based* reward functions, where standard feedback is calculated via token-level labels, and count-based feedback is calculated solely by comparing the entity counts per class between the predictions and ground-truth labels. The results indicate that learning sequence labeling tasks, such as Named Entity Recognition, with aggregate feedback is feasible, even from count-based annotations. Furthermore, our findings suggest that *informed counting* can significantly increase performance.

We acknowledge that the experimental results have potential for considerable improvements, especially regarding the method by which count-based feedback is calculated and attributed to individual label predictions, even when feedback is provided at sequence-level. Although our approach does not completely eliminate the need for labeled datasets, we demonstrate that learning from count-based (or *aggregate*) annotations can achieve reasonable performance for Named Entity Recognition. By proposing this training approach, we are pushing toward more general and less biased annotations, e.g., *counting* instead of *marking* specific entities may lower inter-annotator disagreement. In further studies, the effectiveness of aggregate labels should be explored for more advanced NLP tasks, such as Question Answering or Event Extraction.

## Data Availability

Publicly available datasets were analyzed in this study. This data can be found here: https://huggingface.co/datasets/.

## Footnotes

^1^ https://huggingface.co/bert-base-cased

^2^ https://github.com/chakki-works/seqeval
